# The Lake Superior Region Favorable to Health and Longevity

**Published:** 1855-10

**Authors:** W. B. Herrick


ARTICLE V.—*The Lake Superior Region Favorable to Health and Longevity.* By W. B. Herrick, M. D.

In order to arrive at correct conclusions upon the influence of the climate of any region upon health, it is all important that certain Meteorological phenomena, such as the temperature and barometrical changes, the humidity, density, purity, and other conditions of the atmosphere be closely and accurately observed; and, that the separate and combined influences in modifying vital action, both in health and disease be thoroughly investigated and determined.

A comparison of the following observations made at Fort Wilkins, Keweenaw Point, on Lake Superior, and at Fort Snelling, on the Mississippi, shows, most conclusively, how great are the modifying influences of the large bodies of water in the region of Lake Superior, in tempering its climate:

| | Latitude. | Mean Temperature. | Winter. | Summer. |
|---|---|---|---|---|
| Fort Wilkins, | 47°30′ | 41°46′ | 24°83′ | 58°61′ |
| Fort Snelling, | 44°53′ | 44°8′ | 16°3′ | 71°06′ |
| Extremes at Fort Wilkins, | | | 9°— | 93° |
| " " Snelling, | | | 23°— | 115° |

By the above table it will be seen that at Fort Wilkins on Lake Superior, nearly three degrees north of Fort Snelling on the Mississippi, the mean temperature is nearly the same, but, that it is 8° 53′ colder in winter and 13°9′ warmer in summer on the banks of the Mississippi than on the borders of Lake Superior, and that at Fort Wilkins the thermometer did not at any one time fall lower in winter than 9° below, or rise higher in summer than 93° above; yet at Fort Snelling, it ranged from 23° below in winter, to 115° above zero in summer.

This remarkable difference in the range of the thermometer at two points, nearly in the same latitude, and about the same distance from the coast of the Atlantic, is produced by several causes, the most important of which is, doubtless, that resulting from the fact that but few, if any, bodies of water exist to modify the temperature of the atmosphere of the vast prairies bordering upon and extending hundreds of miles in every direction from the Mississippi, whilst on the contrary, numerous and vast bodies of fresh water abound everywhere in, and surround in every direction the Lake Superior region, cooling the atmosphere by contact, and the absorption of heat by evaporation in summer, and warming it in winter by the liberation of latent heat, during the congelation of vapor and water to form vast bodies of snow and ice. Another reason, not referred to before by writers so far as we know, modifying the temperature of this region, results, doubtless, from the fact, that in the line extending from Lake Superior to the Pacific, nearly in the course of Stevens' Survey, there are but slight if any mountain barriers, to obstruct the free passage of atmospheric currents from the equable and congenial climate of Washington Territory.

Barometric changes as well as those of temperature, influence doubtless the vital functions of animals, to such an extent at least, as to require to be taken into consideration in all investigations regarding the influence of climate upon health. We are not able at this time to avail ourselves of statistics, if any exist, by which to show variations in the pressure of the atmosphere in this region; yet there are certain curious phenomena presented in the sudden rise and fall of water in our lakes, and especially in Lake Superior; which to my mind can be most rationally explained, by supposing that they are the result of varying amounts of atmospheric pressure, acting upon their surfaces at varied times and in different localities.

Concerning these remarkable phenomena, Messrs. Foster and Whitney, in their geological report, published in 1850, make the following remarks:

"Lake Superior possesses all of the sublimity of the ocean. In gazing on its surface, whether stretched out like a vast mirror, reflecting the varying tints of the sky, or ruffled by gently curling waves, or lashed by the fury of the storm, the beholder is alike impressed with a feeling of the grand and the infinite. During a residence of several summers on its borders, our attention has been directed to the fluctuations in the level of its waters; and, while we have failed to detect any ebb and flow corresponding with the tidal action, we have, on the other hand, noticed certain extraordinary swells which appear to be independent of the action of the sun and moon.

"These risings attracted the attention of the earliest voyagers, and they have not failed to record their observations with a minuteness worthy of commendation.

"In the Relation for 1670-71, Dablon uses the following language: 'As to the tides, it is difficult to lay down any correct rule. At one time we have found the motion of the waters to be regular, and at others extremely fluctuating. We have noticed, however, that at full moon and new moon the tides change once a day for eight or ten days, while, during the remaining time, there is hardly any change perceptible. Three things are remarkable: first, that the currents set almost constantly in one direction, viz.: towards lake Michigan, which does not prevent their ordinary rise and fall; second, that they almost invariably set *against* the wind—sometimes with as much force as the tides at Quebec—and we have seen ice moving *against* the wind as fast as boats under full sail; third, that among these currents we have discovered the emission of a quantity of water which seems to spring up from the bottom!' "

Dablon, the writer last quoted, attempts to account in part for these changes by assuming that there are underground passages leading from Lake Superior to the lower lakes. Such a supposition, which, for various reasons, the writer considers untenable, would favor the theory that these changes are produced in whole, or in part, by varying weight of the atmosphere at different times and localities.

The following quotation from the work referred to above, is, as it seems to the writer, nearly conclusive in the support of this view:

"Professor Mather, who observed the barometer at Copper Harbor during the prevalence of one of these fluctuations, has published the results of his observations. He remarks: 'As a general thing, fluctuations in the barometer accompanied the fluctuations in the level of the water; but sometimes the water level varied rapidly in the harbor, while no such variations occurred in the barometer at the place of observation. The variations in the level of the water may be caused by varied barometric pressure of the air on the water, either at the place of observation or at some distant points. A local increased pressure of the atmosphere at the place of observation would lower the water level where there is a wide expanse of water, or a diminished pressure under the same circumstances would cause the water to rise above its usual level.' "

Admitting the correctness of this view, and it would require but a few observations made with the proper instruments on the shores of this or any other lake to test its truth or fallacy, and we have the reciprocal action of the water below upon the atmosphere above, acting in such a manner as to modify the pressure of the atmosphere, making it greater or less according as the water is higher or lower; thus making the action of the lakes in this region important agents to modify barometric pressure as well as temperature; and, additional contributors to health and longevity, by preventing, measurably, those excessive barometric changes so detrimental to life and health.

The hydrometric condition of the atmosphere of this Lake Superior region, is so far as we know, a subject still waiting for and requiring investigation, hence we will state briefly what are some of its geographical and geological features, which must, in conformity with well known natural laws, have the greatest influence in modifying the hydrometric condition of the atmosphere. The numerous bodies of water which surround Lake Superior in every direction, added to its own broad expanse, present an evaporating surface to the sun and air, such as must facilitate to a remarkable degree the accumulation of watery vapor in the atmosphere of this region, yet there are good reasons for believing, as seems to be the general impression of the inhabitants here, that extremes of humidity as well as dryness seldom, if ever, occur. Among the most important modifying influences affecting atmospheric humidity is, doubtless, the charater of the country bordering upon the coast. Upon this subject Prof. Agassiz remarks as follows:

"The general shape of Lake Superior is that of a crescent. But it would be a great mistake to suppose it bounded by curved lines. Its shores are combinations of successive sets of straight parallel lines, determined in each instance by a peculiar system of trap-dykes. These dykes have five general directions, and the outlines of the shores are determined by their combinations."

The mountains and highlands of this region are not elevated to a height sufficient to lower to any great extent the temperature of the atmosphere passing over them, yet their broken and irregular outlines as well as the angularity of their junction one with another constitute numerous projecting barriers imperfectly separated by corresponding depressions, by which all atmospheric currents are broken up and changed in their direction to such an extent that the unfortunate coaster frequently finds himself suddenly checked in his own onward course, and his sail flapping between two atmospheric currents, coming from opposite points of the compass, or what is less common, perhaps he finds himself suddenly drenched by rain or enveloped in fog resulting from the sudden condensation of watery vapor, previously suspended in one or the other of two atmospheric currents, uncongenial both in humidity and temperature, which have suddenly and unexpectedly met, in their passage to and from different elevations of old Superior's castle-walled and terraced coast. In view of the facts here presented, it is evident that in an atmosphere in which the currents are so varied and unlike in direction, temperature and humidity, no great excess of watery vapor can be accumulated or remain suspended for any length of time, without being affected by one or the other of these numerous condensing causes.

Another influence which is very efficient, doubtless, as a preventive to the excessive accumulation of watery vapor in the atmosphere of this region, is the remarkably low temperature of the water of the lake. An accurate and careful experimenter states that he found the temperature of the water of Lake Superior, during the summer, a fathom or two below the surface, but a few degrees above the freezing point.

This low temperature of the water has an important influence upon the atmosphere both in directing its current and modifying its humidity. A well known atmospheric phenomena of this region, is the land breeze, caused doubtless by difference of temperature in the atmosphere over the water and upon the surrounding highlands, by which an almost constantly recurring nightly current is produced of the more dense air of the mountains, towards that which is comparatively rare covering the lake.

With regard to the purity of the Lake Superior atmosphere, it may be stated in brief, that the sources from which the deleterious gases of regions less favored in this respect, originate, do not exist in this region.

Its geological formations being as they are for the most part primitive; and the comparatively low temperature of the atmosphere, are circumstances which preclude the possibility of a generation of gases from decomposing organic or other matter sufficient to change it in any perceptible degree from its normal constitution.

Its purity is also manifest in its invigorating effects upon man and other animals; as also in its remarkable transparency, manifested in the extent of the field of vision, and the softened translucence of a Lake Superior sky.

The above facts being such only, as a recently inhabited country affords, are necessarily few in number, and imperfectly classified, yet the writer believes they are sufficient to justify the following conclusions, in regard to the temperature, humidity and purity of the atmosphere of this country. First, as regards temperature, it is evident that the average amount of cold during the year, and the extremes of heat in summer, and of cold in winter, are less, than in any other region in this country, east of the Rocky Mountains; in the same latitude either east or west.

Secondly, the facts and circumstances which have been mentioned, as having the most influence in determining the amount of pressure exerted by the atmosphere at different times and places, are such as to indicate that barometric changes are never excessive.

Thirdly, it is evident that the vast bodies of water in this region, furnish such a widely extended evaporating surface, as to favor a suspension, in the atmosphere of an excess of watery vapor; yet the causes which have a tendency to produce condensation, are so varied and numerous as to effectually prevent frequent or long continued excessive humidity.

In conclusion, we will simply state, that it must be apparent to all, that no causes favorable to the production of impurities in the atmosphere of this region exist, therefore no argument is required upon this point.

The inevitable conclusions to be drawn from the facts and reasons thus briefly and hastily adduced, are that the region of Lake Superior is one highly favorable to the preservation of health and long continuance of life; being as it is, well adapted, both geographically and geologically to the preservation if not to generation of animals; and having as it has, a pure atmosphere, in which the thermometrical, barometrical, and hydrometrical changes are never excessive.

